# Case of Vicarious Menstruation

**Published:** 1867-04

**Authors:** 

**Affiliations:** Ayr.


### SELECTIONS.

---

### *Case of Vicarious Menstruation.* By Dr. Mason, Ayr.

(Edinburgh Medical Journal, September, 1866.)

Dr. Mason places on record the following singular case: "About the middle of March of the present year, I was requested by my friend Mr. Halden to see a patient whom he had been attending for two or three weeks, but from illness was unable at the time to continue his visits. The patient is a young lady, fifteen years of age, residing at a boarding school in this town; her native place being Liverpool. On calling, I was furnished by the lady of the house with some of the previous history of the case, with which I think it would be better to begin.

"When eight years of age, Miss ——— first began to menstruate, and continued to do so regularly until eleven; menstruation then ceased, and did not reappear until she was thirteen, since when up to the middle of February, 1865, it continued regularly. At that time Mr. Halden was requested to see her, and found what appeared to be a large abrasion of the cuticle in the middle of the right cheek, suppurating in the centre, and inclining to bleed towards the circumference. This sore was exceedingly obstinate, refusing to yield to the local and constitutional treatment resorted to. As far as I can gather, dilute nitrite of mercury ointment, caustic, &c., were applied, and cod-liver oil and iron exhibited internally.

"During the summer, Miss ——— went to Liverpool, her face still unhealed, and, I believe, menstruation very irregular. She was then attended by a medical gentleman; but her face continued so bad, that she did not return to Ayr until the winter. Her medical attendant in Liverpool used locally a solution of sulphate of copper, and covered the part with goldbeaters' skin. Of his constitutional treatment and other *local* applications, I am not prepared to speak with accuracy, as the young lady could give me no clear account of what had been used. From the time her face healed (which I think was in October) until I saw her in the following March, she menstruated every month, the discharge lasting six days each time, and being profuse.

"When I saw her she had a large *patch* on her right cheek close under the lower eyelid, and extending from the outer border of the maler bone to the side of the nose, and about three-fourths of an inch in breadth. On examining it, it appeared as though the cuticle had *melted away*, and numerous little specks of blood were seen on the surface, which was quite wet with a thin serous discharge. An hour before I came, she exclaimed, 'Oh, I feel another place on my face again,' and *immediately* the above appearance was observed. The occurrence of these patches is accompanied by a severe burning pain in the part, lasting for two or three hours. Until very lately, she had not the slightest intimation beforehand that another place was about to break out; the suddenness with which they appeared being almost incredible. Latterly, I observed her lean her head upon her hands, and wear an almost anxious look; and on questioning her, she said she felt rather giddy, and in a quarter of an hour or less another place would break out. It is remarkable that these outbreaks *generally* took place about the same time each day—eleven A. M. Sometimes they occurred in the afternoon, but *by far* the majority at the time specified. As each day almost some new patch occurred, I was very anxious to be present at the time they occurred, and learning the regularity with which they appeared at eleven in the forenoon, timed my visits accordingly. The next day, as I was dressing my patient's face, she exclaimed, 'Oh, I feel a place on my arm.' I at once turned up her sleeve, and there was a large oval patch, fully two inches in length, and one in breadth, on her left forearm, presenting the usual appearances. Here I should mention that these patches assume two different aspects at the outset; sometimes the one and sometimes the other obtaining. The one at the outset appears like a dew of blood, the other has a greater tendency to a serous discharge ending in suppuration. Those that bleed most heal the soonest. But before the places heal (which generally takes place in five or six days) both suppuration and hemorrhage often occur in the same place.

"The hemorrhage, I should observe, does not consist merely ot the dew of blood referred to—*that* is only at the outset—but it is actual bleeding as from a cut, the blood sometimes streaming down the face or other part attacked. The worst place she ever had was on the chin: it did not heal for nearly four weeks, and suppurated freely, the bedclothes in the morning being often soiled by the discharge, but it also at times bled considerable. As soon as one place was healed, it broke out in another, or in the same place over again, some of them having occurred in the same place four or five times. It were tedious and useless to describe all the places that were affected, as all were so similar; suffice it to say, that her face was covered, her chest twice attacked, and both arms and legs.

"For some time I was much at a loss to satisfy myself as to the true nature of the case, but finally came to the conclusion that it was vicarious menstruation. During the course of her attack, I sent Miss ——— into Glasgo to see Dr. M'Call Anderson, and he formed the same opinion of the case as myself, and kindly suggested to me, in a letter subsequently, some alterations in the treatment, to which I shall presently allude.

"While still suffering from the complaint, Miss ——— had a severe attack of whooping-cough, which seemed greatly to aggravate the patches on her face, causing them to bleed freely. This, I have no doubt, was caused by the mechanical exertion during the paroxysms of coughing, sending the blood to the face. At this time also she had frequent and copious epistaxis, generally after a fit of coughing, or after the retching thereby induced; and this *somewhat* relieved the parts attacked.

"A few words now as to the treatment. When I saw Miss ——— she was then using the solution of sulphate of copper to the original spot in the centre of the right cheek, but had not yet applied anything to the new place which had just appeared an hour before my visit. I sent for some oxide of zinc powder, and dusted it well over the part affected, and then covered it with goldbeater's skin. To the original sore I continued the solution, and so could compare the effects of the two applications. The solution caused a good deal of smarting, which continued for some time after its application; but no inconvenience was experienced after the use of the powder. I tried the solution to some new parts, but it only seemed to aggravate them. The original sore was, however, healed by it; but this part, from the first, differed from all the subsequent ones, as it penetrated much deeper, and suppurated very freely for a long time; it is the only place where any sear is left, and it is trifling. Each morning I removed the goldbeater's skin that I had applied the previous day, and, after bathing the part with tepid water, carefully removed the scabs that had formed, so as to prevent the occurrence of cicatrices. The places that appeared on the chest and arms I treated somewhat differently. On their appearance, I bathed them with cold water, and then applied glycerine, and dusted the oxide of zinc powder over it, so as to form a crust; the arms were then loosely bandaged. This plan succeeded admirably on the arms and chest, but did not answer well on the face. Very few scabs formed on the patches on the arms, and they did not bleed so much as those on the face, and healed much more rapidly. The parts affected on the legs bled freely.

"Internally, she got cod-liver oil and the muriated tincture of iron, with liquor arsenicalis. Aloetic purgations were also exhibited, so as to keep the bowels freely open, especially at the time that *any* appearance of menstruation occurred. A hot mustard hip-bath and leeches to the inside of the thighs were employed at the suggestion of Dr. M'Call Anderson, and I think with much benefit.

"In conclusion, let me very briefly recapitulate some of the most striking points in this case.

"In the *first* place, we notice the very peculiar appearance presented by these spots; the thin serous discharge with numerous specks of blood seen in some of them; and the copious dew of blood, followed by actual hemorrhage in others.

"*Secondly.* The instantaneousness of their appearance; the skin appearing perfectly whole and healthy one second, and melted away and bleeding the next—it being only lately that any giddiness betokened their appearance.

"*Thirdly.* The almost uniform regularity with which they occurred, about eleven every forenoon.

"*Fourthly.* The pertinacity with which patch after patch succeeded one another, and the obstinacy with which they so long refused to yield to the influences of remedies.

"Miss ——— has now been quite free from any spots for about six weeks, and no traces of them are to be seen, except when she gets heated or excited, and then the parts that have been attacked look very red. The original spot has left a small depression, but little noticed. And now comes a singular fact, and that is, that although healed and apparently well, her menstruation is not yet properly established.

"During the period that I was attending her, she menstruated *one day every week* for four weeks, there being, however, very little appearance. Then a fortnight would intervene without any menstruation, and then it would begin again as before. And now that she seems perfectly well, I learn that the menstruation is still being carried on in the same manner, the discharge, however, each day of its occurrence being more copious. She is still continuing the cod-liver oil, and has resumed the iron and arsenic, which had been omitted for a short time. On calling two days ago, I was told that Miss ——— had felt dizzy, and that some of the old spots on her face were looking red and angry; I accordingly ordered leeches to the insides of the thighs, and the threatened attack seems to have passed off. But until regular menstruation be established, I shall not be surprised at a recurrence of the attack."—*Half-Yearly Abstract.*

